# The Relative Contributions of Traits and Contexts on Social Network Learning

**DOI:** 10.1162/OPMI.a.31

**Published:** 2025-09-09

**Authors:** Ameer Ghouse, Raphael Kaplan

**Affiliations:** Department of Basic Psychology, Clinical Psychology & Psychobiology, Universitat Jaume I, Castelló, Spain

**Keywords:** memory, cognitive map, social network, learning, contextual memory, personality traits

## Abstract

Navigating the social world is guided by remembering which people know each other. Yet, different factors might influence how social relationships are remembered, where people’s shared attributes could distort a social network’s mnemonic representation. Here, we study whether dyadically shared contexts and personality traits impact how people remember relationships in social networks. Through varying levels of network topological complexity, we find the contexts where people know each other are most memorable and that better contextual retrieval predicts relationship recall. In contrast, shared personality traits affect relationship recall differently depending on social network complexity, where shared negatively valenced traits relate to worse relationship recall in the simple network. Subsequent modeling revealed that as networks become more complex, relationships between more centrally positioned individuals that share negatively valenced traits are better recalled compared to less well-connected individuals. These results suggest contextual memory can serve as a scaffold for remembering relationships in a social network, while affective traits’ impact on social network retrievability depends on emotional valence and the individuals involved. More generally, our findings give insight into how the same social network can be represented differently based on one’s past experience.

## INTRODUCTION

Navigating the social world hinges on drawing upon past experience to remember which people know each other (Schafer & Schiller, 2018; Tavares et al., 2015). Mounting evidence points to episodic memory processes facilitating social network learning by accumulating schematic knowledge from everyday events (Arzy & Kaplan, 2022; Bein & Niv, 2025; Mar & Spreng, 2018). Yet, it is unclear whether other mnemonic details, beyond whether people know each other, informs the learning of relationships that comprise a social network. Notably, long-term memory is susceptible to subjective biases like emotional states making events more or less memorable (Bisby & Burgess, 2014; Kensinger et al., 2006; Madan et al., 2014, 2017; Palombo et al., 2021), and more salient or rewarding stimuli being easier to recall (Shohamy & Adcock, 2010). One possibility is that shared attributes like the context where people know each other (e.g., work, school, etc.), or personality traits (e.g., whether both people are outgoing, obnoxious, etc.) might bias how well a relationship is remembered in a social network (Schwyck et al., 2023). Still, there is a dearth of literature comparing how different event details like affective or contextual information might interfere with, or enhance, retrieval of learned relationships in social networks.

At its core, social network learning involves assimilating how different people relate to each other (Weaverdyck & Parkinson, 2018). A prominent format for representing the relative positions of stimuli in a metaphorical or physical environment are cognitive maps (Tolman, 1948). Cognitive maps are an essential tool for learning spatial environments (Epstein et al., 2017; Tolman, 1948). Notably, much of the same neural circuitry and memory processes used for navigating physical spaces is conserved when exploring abstract knowledge (Aronov et al., 2017; Behrens et al., 2018; Bellmund et al., 2018; Kaplan et al., 2017; Tolman, 1948; Wood et al., 1999), including social networks (Park et al., 2020). Yet, it remains unclear how a propensity away from, or towards specific attributes during learning could influence map-like representations. The relative weighting of one axis or landmark can distort/overrepresent different locations on a spatial map (Tversky, 1981), which could also occur in mnemonic representations of social networks. Normally, cognitive heuristics that differentially weight attributes are exploited to minimize uncertainty (Tversky & Kahneman, 1974). However, whether long-term memory processes draw upon similar heuristics when learning social networks is unclear. If they do, it raises the question which type of event details might influence how social networks are represented?

One domain that could affect how social networks are learned is affective information, where emotional content impacts long-term memory in a myriad of ways (D’Argembeau & Van der Linden, 2004; Hamann, 2001). In particular, emotional content has enhanced saliency (Phelps & Sharot, 2008) along the dimensions of arousal and valence (Brown & Kulik, 1977; James, 1890). The intensity of emotional content can enhance memory performance in amnesics (Hamann et al., 1997), potentially due to its enhanced salience or vividness (Talmi, 2013). However, emotional content has a heterogeneous effect, where its ability to bind associated event details is inhibited by negative valence (Bisby & Burgess, 2014). Conversely, salient emotional content enhances memory retrieval (Shohamy & Adcock, 2010). Independently of whether emotion enhances or inhibits episodic memory, this body of literature hints that affective information might bias mental representations of social networks (Morelli et al., 2017). However, it remains unclear what influence shared affective attributes might hold over social network learning and retrieval.

In parallel, contextual representations are a foundational element of long-term memory that can be expressed in several ways. In its most literal sense, a context can be the spatial environment in which an event takes place (Robin & Moscovitch, 2017). In a more abstract sense, contextual representations can be framed as reflecting long-standing semantic and source associations (Howard & Kahana, 2002; Polyn et al., 2009; Weis et al., 2004), especially in free recall (Howard & Kahana, 2002). Yet the potential physical and abstract aspects of contexts in long-term memory aren’t necessarily mutually exclusive (Ranganath & Ritchey, 2012; Robin & Olsen, 2019). Indeed, when participants are more familiar with the semantic attributes associated with a context, they are more able to vividly imagine a space (Robin & Moscovitch, 2014). Similarly for social networks, both spatial and abstract contexts can potentially assist in inferring whether people know each other. There’s evidence that personal interests (the services a person likes to use) and friendship networks are intricately linked, highlighting how familiar contexts (like being in a running club) can be highly informative of friendships (Yang et al., 2011). Moreover, for spatial contexts, people that live in the same location, or attend the same university are more likely to know each other (Allport, 1954; Arzy & Kaplan, 2022; Gershman & Cikara, 2023). Taken together, this contextual memory work raises the possibility that both spatial and more figurative affiliative contexts might help organize social network representations in memory.

Inspired by the well-studied impact of contextual and affective information on long-term memory retrieval, we investigate how they influence mnemonic representations of social networks. More specifically, we ask whether dyadically shared contexts (Ranganath & Ritchey, 2012; Robin & Olsen, 2019) and/or personality traits (Phelps & Sharot, 2008) are remembered as well as a friendship itself. Furthermore, we’re interested in whether contextual and affective details shape mnemonic representations of social networks. Given the heightened salience of emotional content (Phelps & Sharot, 2008), we hypothesized that shared affective traits would be easier to remember than the friends in a social network. Conversely, better memory for shared contextual details would highlight shared contexts as a key organizer for mnemonic representations of social networks (Schiller et al., 2015). Since emotional details of a long-term memory are known to be strongly linked with other associations (Palombo et al., 2021), we predicted that enhanced recall of shared traits would correlate more strongly with friendship recall than enhanced contextual recall performance. Alternatively, if contextual memory was more predictive of memory for relationships in a social network, it’d be suggestive of contexts potentially serving as a scaffold to remember friendships, similar to the use of memory palaces to organize memories in the method of loci technique (Yates, 1966).

Testing these predictions, we developed an experimental paradigm in which online participants encoded dyadic friendships with a common personality trait and a shared context where the friends know each other (university club). After encoding each dyadic friendship in a social network, participants were prompted to freely associate the previously learned friendships, contexts, and personality traits using a drag and drop test (Kriegeskorte & Mur, 2012). Afterwards, volunteers were administered a surprise associative memory retrieval test in the form of a three alternative forced-choice (3AFC) test separately for shared contexts, personality traits, and friendships. Furthermore, a 3AFC was employed instead of a 2AFC to make it more likely participants engage associative memory processes related to map-like retrieval over recognition memory processes that do not (Schiller et al., 2015). In parallel, analysis of free association behavior in the form of a drag-and-drop task is a powerful tool for studying how participants represent shared personality traits, contexts, and friendships on 2D maps and test whether stimuli placements/relative distances conform to ground-truth specifications (Edelman, 1998; Kriegeskorte & Mur, 2012; Newcombe & Liben, 1982). Therefore, using a free-association drag-and-drop paradigm without any explicit labeling of axes, we can study the latent dimensions (i.e., personality traits or event contexts) that participants prefer to organize social network members. Associative memory tests are well equipped to study how contexts or personality traits shape retrieval of friendships in a social network. Subsequently, cued associative recall can be used to obtain a measure of the ability of participants to remember friendships, personality traits, and contextual information. Additionally, we counterbalanced personality traits for negative and positive emotional valence to investigate potential affective trait valence effects. By using both tasks, we can discover how shared contextual and personality trait information are stored in memory and subsequently influence how social networks are remembered.

Here, we present data from two preregistered online experiments where participants recalled social network members’ friendships, their shared personality traits, and the context where they know each other. In Experiment 1, we studied a simple social network where network members are organized in a single cyclical ring topology. In Experiment 1, participants studied a simple cyclic ring network topology comprising eight university students for which each student in the network had two friends for which they shared a personality trait (emotional) and a university club (contextual) where they met. The experiment consisted of a single run with three blocks: an encoding phase, a drag-and-drop phase, and a 3AFC retrieval phase. In the encoding phase, participants were shown friendship relationships with shared traits and contexts as single sentences. The drag-and-drop phase prompted participants to place the university students such that nearer placements on a rectangular box represented higher likelihood for being friends. The 3AFC test examined recall for each university student’s friends, personality traits, and university club contexts. Additionally, there were two participant pools to test for manipulations of whether personality traits and contexts were correlated with the graph distances on the underlying social network, or whether they were not correlated with graph distances. This manipulation was performed to test the robustness of observed effects.

In Experiment 2, participants performed the same task, but with a more complicated social network, comprising a two cyclic ring topology with a student bridging the two rings. This was a manipulation to test whether results obtained in Experiment 2 would replicate as social network complexity increases. More specifically, finding shared contexts were better remembered than personality traits in Experiment 1, we preregistered that participants in Experiment 2 would better remember contexts. We also predicted that better contextual retrieval would uniquely yield better friendship recall, echoing a contextual memory facilitation hypothesis (Ranganath & Ritchey, 2012; Robin & Olsen, 2019).

Lastly, we developed a computational model to relate drag-and-drop placement data to 3AFC retrieval performance. Consequently, the computational model allowed us to examine how participants’ representation of a social network is reflected in their social recall. We expected that the fidelity of context and trait drag-and-drop 2D representations would predict friendship recall accuracy. By merging computational modeling with two experiments that involve learning social networks of varying complexity, we can gain insights into how knowledge of dyadically shared contexts and traits influences memory for relationships in a social network.

## GENERAL METHODS

### Participants

For all studies, native English speaking participants between 18 and 40 years old (30.3 ± 6.1) from the United Kingdom were recruited using the Prolific platform (Prolific Academic Ltd., 2014). All study participants were compensated and gave informed written consent for their participation. The study was approved by the local research ethics committee (Application number CEISH/29/2022). The study was conducted in accordance with Declaration of Helsinki protocols. Gender ratios were ensured to be evenly split between people identifying as male or female for each experiment. We screened to ensure participants had normal or corrected-to-normal vision, were without any previous neurological or psychiatric disorders, nor had already participated in our previous studies. Experiment 1 attempted to reach a minimum recruitment criterion of 116 participants after excluding participants that couldn’t pass an exclusion criterion to achieve statistical power of 95% given a moderate effect size of *f*^2^ = 0.15 when performing a two-way ANOVA with 3 retrieval conditions as according to the G*Power software (Erdfelder et al., 1996).

### Statistical Analysis

In Experiment 1, a 2-way ANOVA was performed to determine whether within-subject factors such as friendship, personality trait, or context recall performance was significantly different from the mean recall performance and whether this difference depended on the between-subjects factors of whether attributes in the friendship network were correlated or uncorrelated with network distances. We then ran a repeated measures ANOVA to do pairwise comparison of the within-subject factors to determine whether any were significantly different, with a post-hoc analysis of paired *t*-tests with Bonferroni corrections to determine which of them were indeed different. According to our preregistration, we hypothesized that trait recall would be greater than context recall. A second 2-way ANOVA was performed to determine whether context or trait recall performance, or their interaction, predicted friendship recall, and whether this effect depended on social network structure, i.e., whether the underlying network being studied had attributes with semantic similarity correlated with network distances. We preregistered the hypothesis that friendship recall would only correlate with trait and context recall in the participant pool encoding the correlated network structure. Finally, a mixed effects logistic regression model with random intercepts for participants was used to determine whether the valences of the two associated personality traits for a student predicted recall performance for that student.

In Experiment 2, we attempted to replicate the ANOVA results of Experiment 1, except with 1-way ANOVAs given there was only 1 participant pool–i.e., the uncorrelated friendship network. This consideration was made when observing Experiment 1 had no main result that depended on whether the friendship network was correlated. According to the preregistration, we predicted that context recall would be higher than trait recall, and that context recall would correlate with friendship recall more than trait recall. Subsequently, the mixed effects logistic regression model was modified to determine whether retrieval of a student’s personality traits were impacted by affective valence and whether they were a central member of the friendship network. The preregistration hypothesized that network members with shared negative valence personality traits would have worse friendship recall performance.

To evaluate participants’ memory accuracy for the underlying social network, we analyzed responses from the drag-and-drop task by comparing the inferred spatial placements to the theoretical structure of the network. Specifically, we quantified accuracy as a function of stepwise social distance. For each node (i.e., target individual), we assessed whether participants correctly placed its direct neighbors (1st-degree connections) in spatial proximity. For longer paths (e.g., 2nd-, 3rd-, or 4th-degree connections), we tested whether participants preserved the correct sequence of relationships—e.g., accurately placing a 2nd-degree connection also implied correct placement of the intermediate 1st-degree node. Accuracy for each path length was thus defined as the probability of reproducing the full multi-step connection correctly. Corresponding chance levels were computed analytically, based on the probability of randomly arranging 7 neighbors around a given node in the correct chained sequence. These chance levels decrease monotonically with increasing path length, reflecting the growing combinatorial complexity of reproducing longer relational chains. We tested above-chance accuracy for each step length using one-sample *t*-tests against these theoretical baselines and reported group-level performance.

## EXPERIMENT 1

Experiment 1 was a preregistered study (AsPredicted #184,138) designed to test the different contributions of contexts and personality traits on participants’ memory of social networks.

### Participant Pool

Participants were allocated into two evenly allocated groups (58 participants per group). One group of participants encoded social networks where neighbors had correlated shared personality traits and club contexts (correlated network); The other group of participants viewed social networks where neighbors did not have a-priori correlated shared personality traits and club contexts (uncorrelated network). The correlation of traits and contexts were validated using GloVe vector embedding representations (Pennington et al., 2014). More concretely, in the correlated condition, personality traits or social contexts were assigned to nodes such that their semantic similarity was positively correlated with network proximity. That is, nodes that were closer together in the network were more likely to be associated with semantically similar attributes. For example, for a random participant, one node might be described as “smiley” and belong to a “running club”, while its immediate neighbor might be “friendly” and in a “yoga club”—attributes that are semantically similar and topologically adjacent. For another random participant, that same node that was assigned “smiley” may now belong to a “math” club, and the immediate neighbor who is “friendly” could belong to an “AI club”. In the uncorrelated condition, we used the same set of traits or contexts but randomized their assignment across nodes, breaking any systematic relationship between semantic similarity and network distance. Furthermore, the names of people also had randomized positions, meaning no two participants necessarily saw the same set of assignments.

### Task

**Figure 1.**
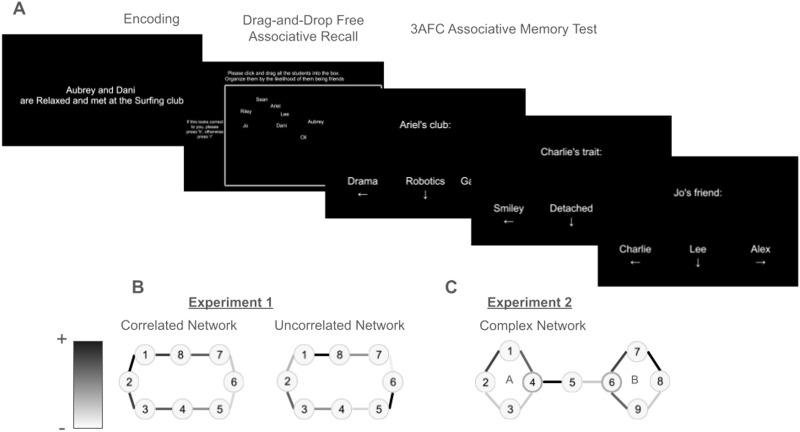
Task design and experimental conditions. (A) Illustration of task blocks. The participants are first instructed to study how students became friends along with their shared personality traits and university club contexts. Then, the participants were asked to drag and drop the students’ names into a rectangular box, where participants were told to arrange students by the likelihood of students being friends. This results in distances representing the inverse likelihood that the students may be friends. Finally, participants were explicitly asked to recall students’ friendships, personality traits, or university club contexts in a three alternative forced-choice (3AFC) test. (B) Experimental conditions. In Experiment 1, 58 participants encoded social networks that were organized where students’ contexts and personality traits correlated with network graph distance–i.e., the friendship network is correlated. 58 other participants encoded social networks that had no organization based on contexts or personality traits–i.e., the friendship network is not correlated. The value of attributes are indicated using the shades of gray on the the edges on the graph, where dark edges would be more similar to other dark edges, while light edges would be more similar to other light edges. (C) In Experiment 2, participants only encoded friendship networks that were uncorrelated. Network members 4 and 6 are central nodes, as indicated by their thicker borders.

All task stimuli were presented using PsychoJS (Peirce, 2007; Peirce et al., 2022) via the Pavlovia platform (PsychoPy Team, 2017). The task consisted of three phases as seen in Figure 1, namely: memory encoding, drag-and-drop, and associative memory recall.

Participants were instructed to read stories about how two fictitious students met, where the story comprises a personality trait that the students share and the context (university club) where they know each other. Each fictitious student had two associated traits, contexts, and friends each. For participants encoding correlated friendship networks, network distances between students correlated with semantic similarity of associated contexts and traits, validated using a global vector word embedding (Pennington et al., 2014). For participants encoding uncorrelated friendship networks, network distances did not a-priori correlate with semantic similarity of contexts and traits. Participants encoded friendships for eight fictitious students. There were three blocks of the encoding trials, consisting of 24 total encoding trials. Each block had an attention check, where the participant had to press the spacebar within 3 seconds in each block after a randomly selected trial. After encoding, the participants performed a drag-and-drop free association task, placing names of the fictitious students into a rectangular box based on the participants’ inferences of friendship likelihood. After the participant confirmed their placements, the participant reported a continuous confidence rating from 1 (not confident)-7 (very confident) for their placement decisions. Their drag-and-drop time was also recorded. If participants idled for more than a minute, they were kicked out of the experiment and their data excluded from analysis. Next, a 3AFC associative memory retrieval test was administered. Each student in the network was prompted as a cue for associative recall. This comprised three conditions: trait, context, and friendship recall. Trials were counterbalanced for conditions. For the eight fictitious students, each student had two friends, traits, and contexts, as a consequence of the ring structure. There were 48 recall trials per block. Participants were given two blocks of recall trials for a total of 96 trials.

An exclusion criteria was imposed to ensure the quality of online participant data included for analysis. The first exclusion criterion for the study was based on participant’s idle time when performing the study. If the participant did not move their mouse for more than a minute during the drag-and-drop phase, they were kicked out of the experiment and excluded from further analysis. Furthermore, there was active monitoring of response times (RTs) during memory retrieval blocks–memory retrieval trials timed out after 10s and if 5 consecutive memory retrieval trials timed out, the participant would have the experiment terminated and their data excluded from further analyses. In Experiment 1, 174 participants completed the study and were remunerated on Prolific, within which 116 passed the exclusion criteria and were included for analysis.

### Results

**Figure 2.**
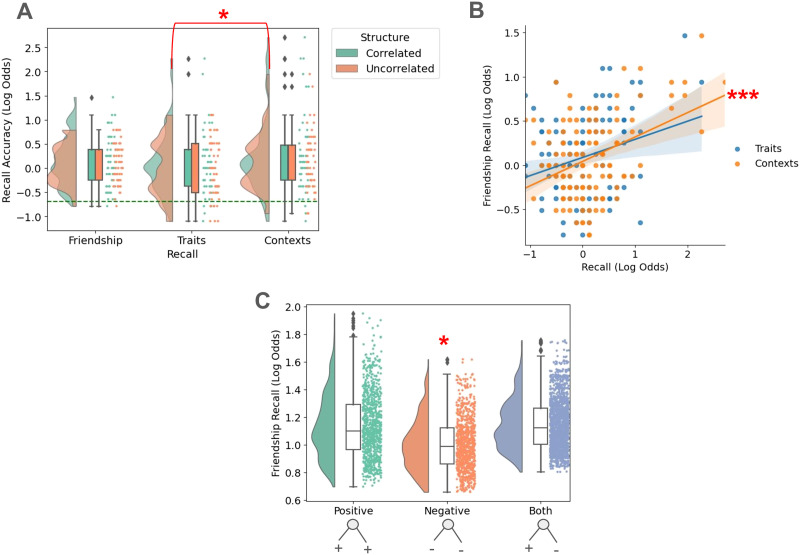
Experiment 1 3AFC recall performance. (A) Average participant recall of network members’ attributes separated by whether participants were encoding networks with correlated or uncorrelated attributes. Context recall performance is greater than recall for friendships and traits, regardless of whether the network structure had correlated attributes. Chance level is indicated by a dashed-green line. (B) Correlations between participants’ friendship recall and context or trait recall. A significant positive correlation was only found between recall of network members’ friends and contexts. (C) Trial-by-trial mixed effect model predictions of friendship recall odds for network members with either positive, negative or both positive and negative personality traits. Recall for a network member’s friend is significantly impeded when the network member only has negatively valenced personality traits. * *p* < 0.05, ** *p* < 0.01, *** *p* < 0.001.

To begin, we analyze the results of the 3AFC associative memory recall test. This analysis provides initial clues to whether personality traits or contexts influences friendship recall. We first perform an ANOVA to test whether friendship, context, or personality trait recall have the same mean performance, as seen in Figure 2A. Contrary to our preregistered hypothesis, the ANOVA revealed that only context recall accuracy (53.0%) was significantly different from mean recall (52.4 % , *F*(1) = 4.97, *p* = 0.027). Using a repeated measures ANOVA, we confirmed that there were within-subject factors, i.e., recall conditions, that were different from all other within-subject factors (*F*(2, 230) = 3.1988, *p* = 0.043). Post-hoc paired *t*-tests revealed that only context recall performance was higher than trait recall performance (*t* = 2.48, *p*_*corrected*_ = 0.043). To accompany this analysis, we studied whether personality traits or contexts facilitates friendship network recall. To that extent, we performed a second ANOVA to test whether context recall, personality trait recall or their interaction predicted friendship recall (see Figure 2B). Only context recall significantly predicted friendship recall (*F*(1) = 10.2432, *p* < 0.01) with a positive correlation (*R* = 0.4, *p* < 0.001). None of the ANOVA results depended on the correlated structure of the friendship network, contrary to our preregistered hypothesis. Informed by the null personality trait results in the previous analysis, we performed a follow-up mixed effects analysis to test whether the valence of associated personality traits for friendship network members impacted their recall of friendships (Figure 2C). We observed that when students had negatively valenced personality traits, participants had worse friendship recall (*Z* = −2.11, *p* = 0.035).

**Figure 3.**
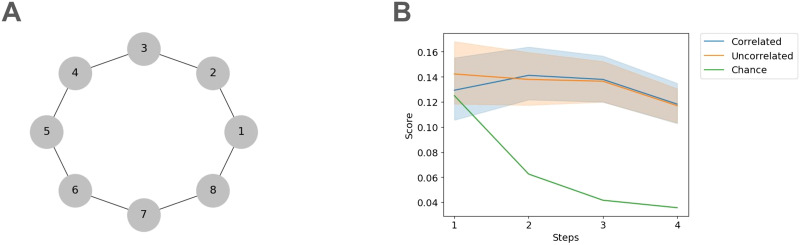
Participants maintain multi-step representations of social networks in Experiment 1. (A) The underlying social network encoded by participants in Experiment 1. (B) Participants’ drag-and-drop placements follow the underlying network graph’s distances for network distances greater than one step. Rank placement accuracy for each accumulative step is above chance.

We then analyze the results of the drag-and-drop task, where participants needed to reconstruct a friendship network with an underlying topology as seen in Figure 3A. For both groups encoding the correlated (Step 1: *t* = 0.29, *p* = 0.385, Step 2: *t* = 6.05, *p* < 0.001, Step 3: *t* = 8.46, *p* < 0.001, Step 4: *t* = 8.46, *p* < 0.001) or uncorrelated network structure (Step 1: *t* = 1.09, *p* = 0.14, Step 2: *t* = 3.45, *p* < 0.001, Step 3: *t* = 9.41, *p* < 0.001, Step 4: *t* = 9.41, *p* < 0.001), participants were able to maintain accurate multi-step representations of the network structure at above chance accuracy (see Figure 3). We provide visualizations of Experiment 1’s drag-and-drop results in the Supplementary Figure S2A–C.

## EXPERIMENT 2

### Participant Pool

Experiment 2 followed similar recruitment criteria as Experiment 1. However, the size of the participant pool was different, as we were aiming to replicate the results of the uncorrelated network structure only, with more modest expected effect sizes. Concretely, we hypothesized a relatively moderate effect size (*f*^2^) for our primary hypothesis of 0.40 with statistical power of 0.80 when performing a one-way ANOVA with three retrieval conditions, 80 participants that passed the exclusion criterion were recruited for Experiment 2.

### Task

We performed a preregistered online replication (AsPredicted #195,453) of Experiment 1, but manipulated the underlying topology of the friendship network to be more complex. This consisted of making a friendship network with two cyclic cliques comprising four students connected by a bridge student. As a consequence of this topology, one network member in each clique had a third friend corresponding to the bridge student, more than all other network members who only had 2 friends. The higher connectivity of these network members highlight their centrality, or importance in the network (Friedkin, 1998; Paluck & Shepherd, 2012; Sherif & Sherif, 1964; Wasserman & Faust, 1994). We refer to biases with respect to these two central network members as centrality biases. With this network topology, participants studied nine students in total in Experiment 2. All 116 participants in Experiment 2 viewed a friendship network structure where the semantic similarity between two students’ traits and contexts were uncorrelated with network distances, i.e., the uncorrelated network. In this experiment, there were three encoding blocks consisting of a total of 30 trials. Participants then performed a drag-and-drop free association test of the nine students’ friendship likelihoods on a 2D rectangular box, where distances correlated with inverse friendship likelihood, similar to Experiment 1. Finally, two blocks of a 3AFC recall test were administered. Trials were counterbalanced for conditions. The same exclusion criteria applied for Experiment 2. Each block tested associations within each of the two cyclic ring cliques, where each ring consisted of four students who have two friends, traits, and contexts. Furthermore, associative recall of the bridge student between the cliques was tested adding two more friendship, context, and trait recall trials. Consequently, there were 54 trials per block, and ultimately 108 total recall trials in the experiment.

In this experiment, 162 participants completed the study and were remunerated on Prolific, where 80 participants passed the exclusion criteria and were included for analysis.

### Results

**Figure 4.**
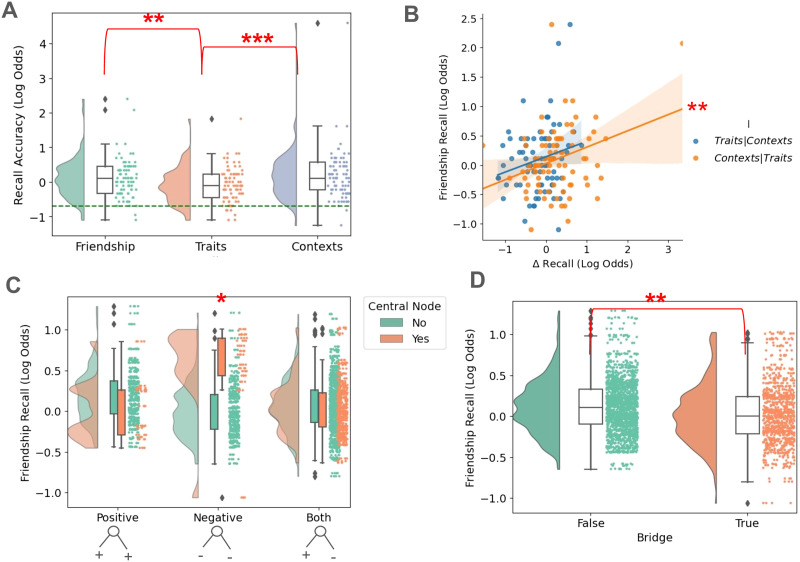
Experiment 2 3AFC recall results. (A) Average participant recall of network members’ attributes. As in Experiment 1, spatial contexts are better recalled than friendships and personality traits. Additionally, we find that recall for traits is significantly less than average recall. Chance level is indicated by a dashed-green line. (B) When performing partial correlations, only context recall is significantly correlated with friendship recall. (C) When we model centrality in the mixed effects model, we recover an impact of negative valence on friendship recall. Central nodes induce greater recall for friendships of students with negatively valenced personality traits. Recall for friendships of non-central students with negatively valenced personality traits however is worse. (D) Recall outcomes for bridge node network members, on the other hand, does not interact with the associated valenced personality trait attributes of the network member. Participants overall have less recall performance for bridge nodes regardless of other factors. * *p* < 0.05, ** *p* < 0.01, *** *p* < 0.001.

In Experiment 2, we determined whether the results of Experiment 1 would replicate in a more complex underlying network. We divided the 8 students into 2 distinct cyclic ring cliques with a new 9th student acting as a bridge connecting the two cliques, but not belonging to either of them. In contrast with the other students, the two students linking each of the two cliques with the bridge student are considered central student network members. First, we replicated the initial ANOVA in Figure 4A, finding recall for context associations (53.6%) is significantly greater than average recall performance (51.0 % , *F*(1) = 6.23, *p* = 0.0132). However, we additionally find that trait recall performance (47.4%) is significantly worse than the average recall performance across attributes (*F*(1) = 7.013, *p* < 0.01). Using a repeated measures ANOVA, we again find that there are within-subject factors that are different from others (*F*(2, 160) < 0.001). Post-hoc paired *t*-tests revealed that friendship recall performance was greater than trait recall performance (*t* = 3.08, *p*_*corrected*_ = 0.009), and context recall performance was greater than trait recall performance (*t* = 3.92, *p*_*corrected*_ = 0.0006). In parallel, we find that both shared context (*F*(1) = 10.7, *p* < 0.01) and trait retrieval performance (*F*(1) = 4.70, *p* = 0.033) significantly predicted friendship recall with no significant interaction. Notably, trait recall correlated with friendship recall with an *R* = 0.404 (*p* < 0.001), while context recall correlated with friendship recall with an *R* = 0.468 (*p* < 0.001). As a control analysis to test the robustness of these correlations, we observed a significant partial correlation of context recall with friendship recall (*R*_*Contexts*,*Friends*|*Traits*_ = 0.312, *p* < 0.01); in contrast, a partial correlation of trait recall with friendship recall was not significant (*R*_*Traits*,*Friends*|*Contexts*_ = 0.205, *p* = 0.06). Taken together, these results recapitulate Experiment 1’s result of contextual attributes providing a more prominent memory scaffold for friendship relationships. Finally, mixed effect models were used to test the heterogeneous effect of personality trait valence on friendship recall (see Figure 4C). We observed that participants have better friendship recall for students with negative personality traits only when they are central in the friendship network. Otherwise, for non-central students we recover the results of negative personality traits interfering with friendship recall (*Z* = 2.02, *p* = 0.044). This highlights how network centrality impacts recall of social networks, especially along the negative shared personality trait dimension. Conversely, for bridge nodes, there were no interactions with the valenced nature of the personality traits; participant recall performance for bridge nodes are generally reduced (*Z* = −2.58, *p* = 0.01).

**Figure 5.**
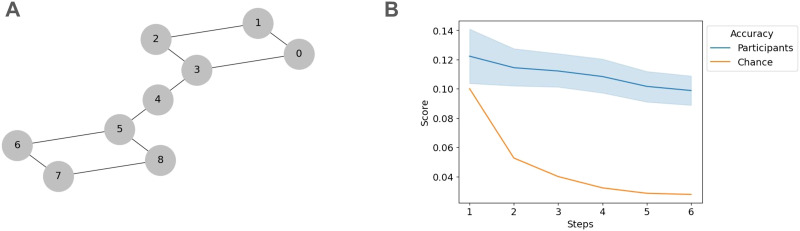
Participants repeated their ability to maintain multi-step representations of social networks in Experiment 2. (A) The underlying social network encoded by participants in Experiment 1. The topology consists of two groups separated by a bridge node (B) Participants’ drag-and-drop placements follow the underlying network graph’s distances for network distances greater than one step. Rank placement accuracy for each accumulative step is above chance.

We then analyzed the drag and drop results to determine whether participants could reconstruct the underlying network seen in Figure 5A. In contrast with Experiment 1, participants are able to maintain accurate placement of friendship network members across all steps (Step 1: *t* = 1.88, *p* = 0.031, Step 2: *t* = 2.81, *p* < 0.001, Step 3: *t* = 7.60, *p* < 0.001, Step 4: *t* = 10.77, *p* < 0.001, Step 5: *t* = 11.36, *p* < 0.001, Step 6: *t* = 11.36, *p* < 0.001, see Figure 5B). Furthermore, it appears that participants collapse the complex topology along the bridge student, whose clique membership is ambiguous, resulting in a simplified network reconstruction. This result reiterates the behavioral recall results seen in Figure 4D. We visualize the average participant placement results in Supplementary Figure S2D.

## COMPUTATIONAL MODEL

### Theory

To model how participants recalled social relationships, we assumed that their memory for links between individuals was influenced by semantic similarity—based on shared traits and contexts—modulated by cognitive biases. Specifically, we fit a model where trait and context similarities were weighted differently depending on their valence (positive/negative) or type (physical/non-physical), using separate exponential bias parameters, *β*. These bias parameters, *β*, control the extent to which each similarity dimension contributes to perceived social connection likelihood. Higher bias values correspond to higher sensitivity to that dimension, and vice versa. The cognitive hypothesis is that memory for social structure is systematically distorted by individual biases toward certain semantic dimensions. For instance, a participant might overemphasize positive trait similarity when recalling who is connected to whom.

In the experiments, participants were instructed to spatially arrange individuals based on perceived likelihood of social connection. We interpret this layout as reflecting internal clustering based on both observed and inferred features (e.g., shared traits, shared contexts). The model assumes that participants are more likely to place individuals near each other if they are semantically similar, but that the influence of each semantic dimension is biased, depending on its valence or category.

Let *D*_*i*_ and *D*_*j*_ be the attribute vectors for students *i* and *j*, respectively, where *D*_*i*_ ∈ ℝ^3^. The first element of the vector is a valence measure of a student’s assigned trait attribute given the word “Smiley” as a reference for peak value, the second element of the vector is a measure of how athletic the university club they attend are given the word “Running” as a reference for peak value, and the third element is a measure of whether the student is a central node in the friendship network (Experiment 2). Similarly, let *C*_*ij*_ ∈ {0, 1}^3^ indicate which bias conditions are shared between them. *C*_*ij*1_ = 1 if the students both have positive-valence personality traits, otherwise they have negative-valenced personality traits. *C*_*ij*2_ = 1 indicates whether the students both attended physical (athletic) university clubs, otherwise they both attended non-physical university clubs. *C*_*ij*3_ = 1 occurs if at least one of the students in the comparison is a central node in the network (Experiment 2 only). The function *f* is a factorized squared exponential function, defined as:

$$f\left(D_{i},D_{j},C_{ij},\beta ,\alpha \right)=\left\{\begin{array}{l} \prod_{k=1}^{3}\text{exp}\left(-C_{ijk}\beta_{k}{\left(D_{ik}-D_{jk}\right)}^{2}-\left(1-C_{ijk}\right){\hat{\beta }}_{k}{\left(D_{ik}-D_{jk}\right)}^{2}\right),\text{if}\;i\neq j\\ \alpha \end{array}\right.$$ (1)

*β*_*k*_ reflects the strength of a bias toward a shared attribute when *C*_*ijk*_ = 1, while $\hat{\beta }$_*k*_ reflects the strength of an opposing bias, i.e., when *C*_*ijk*_ = 0. *β* is defined in ℝ^+^, and can lead to interesting interpretations when performing logarithmic transformations–when a bias *β* is 0, we can say the participant places no distortion of their representational space along this attribute dimension; when it is greater than 0, the participant expands their representational space along this attributes dimension; and when it is less than 0, the participant compresses their representational space along this attribute’s dimension. Finally *α* is a dispersion parameter, reflecting the tendency to assign a person to a singleton cluster (i.e., no close associations). *α* also is in ℝ^+^.

The unnormalized probability of assigning student *i* to the same group as student *j* is proportional to this similarity:

$$P\left(c_{i}=j|D,C,\alpha ,\beta \right)\propto f\left(D_{i},D_{j},C_{ij},\beta ,\alpha \right)$$ (2)

This probability function can then be normalized as follows:

$$\text{Prob}\left(c_{i}=j|D,C,\alpha ,\beta \right)=\frac{f\left(D_{i},D_{j},C_{ij},\beta ,\alpha \right)}{\sum_{n=1}^{N}f\left(D_{i},D_{n},C_{\text{in}},\beta ,\alpha \right)}$$ (3)

This function has three parameters to fit: the vectors *β*, $\hat{\beta }$, and *α*. The set of all parameters is denoted as *θ* and a parameter fit $\hat{\theta }$. We fit this model by minimizing the Kullback-Leibler divergence between the participant’s drag-and-drop behavior and the model’s predictions:

$$\hat{\theta }={\text{argmin}}_{\theta }\left[KL\left(P\left[\text{DragDrop}\right]\|P\left[\text{Model}|\theta \right]\right)\right]$$ (4)

To operationalize participants’ behavior, we treat their drag-and-drop layout as an inverse-distance-based probability distribution: the closer two individuals are placed, the more likely the participant believes they are friends. For each students *i* and *j*, we define the empirical distribution as:

$$P\left(c_{i}=j|\text{DragDrop}\right)=\frac{\frac{1}{d\left(S_{i},S_{j}\right)}}{\sum_{n=1}^{N}\frac{1}{d\left(S_{i},S_{j}\right)}}$$ (5)

where *d*(*S*_*i*_, *S*_*j*_) is the Euclidean distance between students *i* and *j* in the participant’s spatial layout.

In order to fit the parameters, we used a Newton-style optimization with trust regions (Sorensen, 1982).

### Model Comparison and Hypothesis Testing

To test whether participants’ spatial representations were systematically shaped by semantic biases (rather than being neutral or unbiased), we compared the biased model (with distinct bias parameters for each condition) against an unbiased model, which shares a single *β* across all attributes.

This comparison was performed via leave-one-out cross-validation at the participant level. For each participant, we systematically left out one student when fitting the parameters for both models, then evaluated whether the biased model yielded lower KL divergence on the held-out student’s placements than the unbiased model. Across participants, we then tested whether the biased model consistently outperformed the unbiased model. We describe the procedure in further detail in the supplementary material.

Finally, to assess whether individual differences in semantic bias (as quantified by the fitted $\hat{\beta }$ parameters) were related to behavioral outcomes, we performed an ANOVA relating model parameters to friendship recall accuracy. This allowed us to test the broader hypothesis that semantic distortions in network representation affect memory for true social connections.

### Results

We used a computational model fit only to the drag-and-drop data to relate placements with the 3AFC recall task. This computational model lets us examine the dimensions by which participants distort representations of friendship networks and its effect on retrieval outcomes. The cross-validation procedure demonstrated that the biased model better generalized to the data compared to the unbiased model (Experiment 1: *t* = 8.29, *p* < 0.001, Experiment 2: *t* = 2.36, *p* = 0.01).

**Figure 6.**
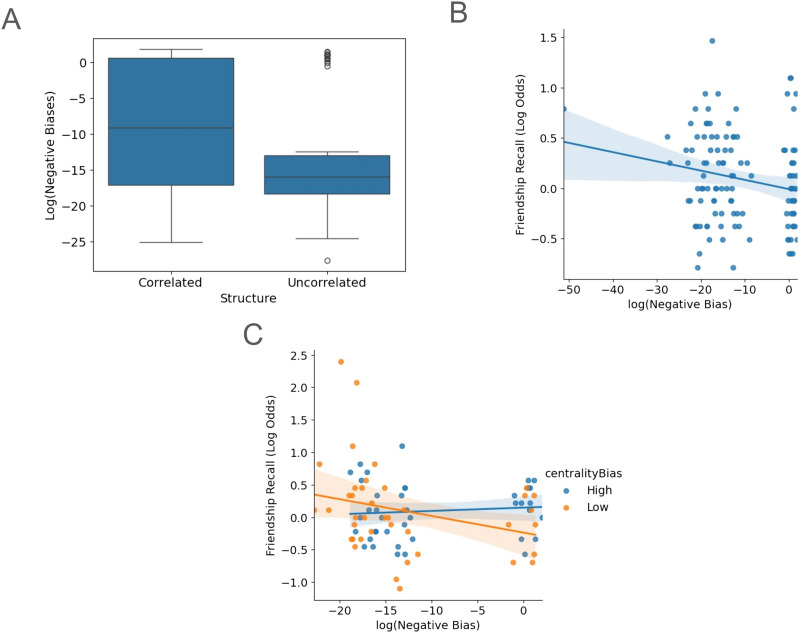
Modeling reveals biased placements along a negative trait dimension affects friendship recall. (A) In Experiment 1, participants encoding friendship networks with correlated attributes had a greater preferences for placements along a negative personality trait dimension. (B) Relating friendship recall with the negative trait bias, we observed a significant negative correlation. (C) In Experiment 2, participants recover the negative correlation between friendship recall performance and placement biases for negative personality traits seen in Experiment 1, only when there’s not a strong bias to make drag-and-drop friendship placements based on central members of the friendship network. Centrality bias is a continuous variable, which we converted into low and high categories with a median split to better visualize the interaction effect.

We first analyzed data from Experiment 1 to determine whether any parameters were predictive of the experimental design (i.e., whether participants were encoding the correlated or uncorrelated network structure). In Figure 6A, we show that biases for drag-and-drop placements along a negative personality trait dimension were greater when encoding correlated networks (*t* = 2.57, *p* = 0.01). However, none of the biases were significantly different from one another (*F*(3) = 0.96, *p* =.41). When relating placement biases to friendship recall, we found that biases for placements along negatively valenced personality traits negatively correlated with friendship recall (*F*(1) = 4.02, *p* =.047, *R* = −0.167; Figure 6B), which suggests that there is a memory interference effect provoked by negative trait content.

Next, we analyzed data from Experiment 2 to see whether the model results replicate for more complex social networks. Again, none of the biases were significantly different (*F*(4) = 0.512, *p* = 0.726). When relating placement biases for negative trait dimensions to friendship recall, we found that we could recover the negative correlation only if we consider the interaction with centrality biases (*F*(1) = 4.71, *p* = 0.033). If participants were not biased towards placing students near central students, we recovered the negative correlation (Low centrality bias: *R* = −0.283, High centrality bias: *R* = 0.13). This highlights the nuance by which network topology impacts friendship network distortions.

## DISCUSSION

In light of recent work showcasing how humans maintain mental representations of social networks (Arzy & Kaplan, 2022; Park et al., 2020; Son et al., 2023), we studied how various attributes linked to network members, namely contexts and personality traits, impact the structure of these mental representations. We ran two preregistered experiments that varied the complexity of friendship networks to test if results were generalizable across differently structured networks. Across all experiments, participants were prone to relate contexts with friendships, providing evidence that contextual versus affective information facilitates memory of social network structure. In contrast, the polarity of shared personality trait valences had a heterogeneous effect on the participants’ ability to recall friendships in a social network, where this relationship was sensitive to the structural complexity of the network. In what follows, we discuss the implications of our findings on learning social networks and explore how biases for specific attributes can distort how people represent relationships between individuals.

Our finding that contexts helping to facilitate memory for friendship relationships is in line with previous work in other domains (Ranganath & Ritchey, 2012; Robin & Olsen, 2019; Robin et al., 2016). Furthermore, as participants are vividly able to imagine contexts, they subsequently are more able to recall other associated elements in source memory (Robin & Moscovitch, 2014). Experiment 2 appeared to show personality traits may offer a similar scaffold as contexts, but regressing the effect of contextual recall from personality trait recall annulled personality trait recall’s correlation with friendship recall. This demonstrates any correlation was merely coincidental with contextual retrieval. This finding could imply that recalling contexts not only facilitates social network recall, but could serve as a general scaffold for recalling the associated attributes of network members. However, we caution explicit causal interpretation of these results, as other evidence points to recollection of social relationships driving recall of other social attributes (Jolly et al., 2023). In the context of our computational modeling results, participant variations in how they prefer to bias their placements with respect to contexts do not impact recall of friendships. These modeling results imply that distortions of mental representations along contextual dimensions have minimal impact on downstream associations. The lack of a context impact on downstream associations may have immediate consequences with regards to evidence integration and decision making in the sense that, as humans explores a state-space, they will rationally encode social relationships–they are not perturbed by the nature of the context. Yet, the contexts in this task are abstract in the sense that they are merely the names of university clubs where fictitious students meet. In other words, we present context as a set of discrete, noncontiguous, entities. However, contexts, in the sense of space, in the real world have contiguity (Maurer & Nadel, 2021) with various attributes such as boundaries and (Shelton & McNamara, 2001), landmarks (Lew, 2011), that impact how encoded content is prioritized. All these attributes are known to have a privileged role in the brain’s contextual processing system, particularly the hippocampal formation. Similarly, the contexts presented in this study carried not just spatial, but also semantic information, corresponding to network members’ shared interests (e.g., people interested in math go to a math club). Therefore, it’s difficult to unpack whether the contextual prioritization observed here is semantic or spatial in nature. Contextual cues as presented in this study elicit anterior hippocampal activitation (Ritchey et al., 2015), corresponding to a potential gist-like representation of contexts (Robin & Moscovitch, 2017), whereas more vividly recalled scenes would correspond to more posterior hippocampus activity (Zeidman et al., 2015), attesting to differential processing, and speculatively differential prioritization, for semantically tied (gist) spatial associations compared to more vivid physical spatial associations. Consequently, to determine the viability of spatial scaffolding for encoding friendships, future studies can test whether prioritization of friendship encoding occurs at salient points in a navigable contiguous map and further differentiate between spatial and categorical contextual mnemonic scaffolds.

The affiliative contexts we use in our paradigm are different from the purely spatial contexts (Robin & Moscovitch, 2017) or long-standing source associations (Howard & Kahana, 2002; Polyn et al., 2009; Weis et al., 2004) typically used in contextual memory paradigms. Consequently, we can’t definitively say contextual memory effects of shared club membership are due to assumed direct learned associations, or if shared club membership provokes spatial imagery that facilitates encoding (Hassabis & Maguire, 2007; Robin & Moscovitch, 2017). In either case, our results implicate contextual details in adding structure, be it causal or relational, between different individuals in a social network. Notably, any type of contextual scaffolding of learned complex associations would allow participants to structure various disparate communities like family trees, religious fellowships, or formal organizational charts in memory (Brewer, 1995; Park et al., 2020). Moreover, learning a latent graph structure to link different stimuli is a key element of cognitive map-like representations (George et al., 2021; Mark et al., 2020), which is consistent with the putative role of cognitive maps in guiding social network learning (Arzy & Kaplan, 2022; Schafer & Schiller, 2018; Son et al., 2021). Hence, we anticipate that the contextual scaffolding effects observed here would be replicable using any event context that possesses a time (when), location (where), and semantic (what) association (Buzsáki et al., 2022; Heald et al., 2023), even if some of those dimensions aren’t explicitly presented in our study. Still, further research is needed to determine whether other putative contextual task attributes like reward, attention, task demands, or goals (Dimsdale-Zucker et al., 2023) would also yield facilitatory effects when retrieving relationships in a social network.

In contrast with contexts, affective content (personality traits) heterogeneously affects memory of friendship networks. In a mixed effects model, we observed that this lack of synchrony is affected by the valence of personality traits. More specifically, a network member having negatively valenced personality traits interfered with the participant’s ability to recall the network member’s friends, which echoes memory interference effects observed in previous work (Bisby & Burgess, 2014; Bisby et al., 2018). Additionally, if network members had both a positive and negative trait, participant recollection of this network member’s friends more closely followed recollection of network members with only negative traits. This may demonstrate how the effect of preferences for grouping participants by negative traits overrides the effects of a contradicting positive group label (Lau et al., 2018). Similarly, when assessing the remembered representational structure of the social network in the drag-and-drop task using a computational model, we observed that participants who showed lower bias toward negative trait information—i.e., who compressed representations along that dimension—tended to have better recall of the underlying network. This suggests that deemphasizing negatively valenced traits may allow for more effective integration of other, structurally relevant cues such as affiliative context. In this way, the model provides a mechanistic account of how certain kinds of information (e.g., traits vs. context) may distort or support memory for social relationships by modulating the geometry of internal representational space. Curiously, whether negative personality traits interfere with friendship recall depends on the network centrality of the student. Participants’ friendship recall improves for central members with shared negative personality traits, while participants have worse recall when recalling friends of non-central members with shared negative personality traits. We speculate that this could be due to the coinciding saliency of a central network member along with the negative personality trait. As a network member is presented more in encoding trials due to their central nature, it also leads to prioritized encoding (Mulhall, 1915; Rock, 1957). Additionally, central entities may be perceived as more information rich and therefore more salient (Basyouni & Parkinson, 2022; Jiang et al., 2023; Paluck & Shepherd, 2012). In the present work, centrality was studied using eigenvector centrality, a measure of how well-connected members are connected to other well-connected members instead of other measures of centrality, attesting to the information richness of these identified central network members in this study (Weaverdyck & Parkinson, 2018). Neural evidence further points to centrality as a strong driver of friendship recollection (Jiang et al., 2023; Schwyck et al., 2023). Centrality interacts with personality traits, impacting who people view as central entities: for example, in networks based on trust, people seek more empathetic people as central entities, while in networks based on fun people seek others with high well-being as central entities (Morelli et al., 2017). Negative content tends to be rated as more arousing (Lang et al., 1993), further attesting to its saliency (Sutherland & Mather, 2012). Therefore, both centrality and negative affective content may hold a unique role in strengthening memory for friendships.

We ran a mixed effect model for predicting both trait and context recall performance, and found no significant impact of either network centrality, nor trait valence on predictions. This indicates that attributes primarily assist in maintaining representations of connections between network members, but not necessarily recalling the attributes themselves. Speculatively, this may be due to the task structure, where participants were informed of the necessity to remember friendships in the instructions. Alternatively, understanding social relationships may itself be rewarding like money or food (Baumeister & Leary, 2017). Also, social concepts themselves can make complex learning easier (Hackel et al., 2024), suggesting that the mere presence of social attributes can potentially facilitate the complex relational learning necessary to remember a social network.

In both experiments, the participants were able to accurately place the students in the friendship network in its ground truth topology. This illustrates that distortions, predispositions to not directly relevant attributes when making joint relationship likelihood judgments for network members, does not interfere with the ability to make accurate judgments. The ability to make accurate likelihood judgments across multiple steps recapitulates previous results on the use of cognitive maps in social navigation (Son et al., 2024). This social navigation model leverages temporal dynamics of experience to form networks (i.e., graphs to learn how to connect distant network members), which implies that biases may similarly be dynamically updated through experience, and might not be a fixed value like we modeled here. Understanding the intrinsic link between the biases that distort mental representations of social networks alongside the actual construction of a social network graph would be instrumental to understanding how subjective distortions emerge, whether due to egocentric biases (Rodríguez Aramendía et al., 2025; Todd & Tamir, 2024) or other heuristics. Indeed, the temporal manner by which we navigate graphs give rise to understanding community structures in networks (Schapiro et al., 2013). One possibility is that certain attributes of network members modulate the discovery of these communities, affecting subsequent inferences when interacting with network members. Understanding how these processes interact can be a crucial step towards understanding individual differences when making inferences on social graphs.

Here, we varied social network topological complexity, uncovering contextual and affective biases. Notably, network size is another important factor that can affect mental representations. The inherent increase in memory cost as networks scale up would demand resource rational behavior (Griffiths et al., 2015). Many heuristics can be implemented to alleviate this memory cost, such as decomposing networks, or chunking (Pothos, 2007). Neural evidence suggests events that occur closer together tend to cluster together in memory (Schapiro et al., 2013). Furthermore, theoretical suggestions support the idea of clustering in social networks to facilitate action in such networks, while having consequences for collective behavior (Momennejad, 2022). Leveraging this clustering potential on social networks could theoretically drastically reduce the inferential costs of similarity likelihoods (Hackel et al., 2024), but potentially give rise to stereotyping and prejudiced behavior (Allport, 1954; Gershman & Cikara, 2023). Given the theoretical prominence for how humans leverage reductions and simplifications of real relationships in social learning (Hackel et al., 2024), the manner by which participants distort their representations of social networks may similarly be modulated. Indeed, our drag-and-drop results alluded to the possibility that participants learn the cliques of the social network in Experiment 2, providing evidence that they may have learned community structures in the network. However, we did not perform a direct analysis of community detection, nor was the task-design designed such that participants should learn communities like previous work with presenting connections on the network graph with a random walk (Schafer & Schiller, 2018). Consequently, we could not analyze if the cliques discovered were due to understanding the clique as a community, or whether participants were performing very well at inferring direct relationships and inferred communities by chance. Nonetheless, our work opens a new avenue to investigate how humans simplify learning of social networks, and how this simplification impacts mnemonics that leverage attributes for recalling a social network.

There is a growing body of literature studying how social attributes contribute to mental representations of relationships and networks (Baek et al., 2021; Falk & Bassett, 2017). Recent work has found that people who are similar are more likely to become friends, which can be due to similarity in individual preferences and/or environmental factors in a phenomenon called homophily (Dunbar, 2018; Schwyck et al., 2023). In parallel, encoding cognitive map-like representations of social features is a putative key building block of learning social networks. However, it isn’t readily apparent how concepts from previous work like social features and homophily relate to the contexts and traits from our paradigm. One possibility is that binding relationships to the multidimensional structure afforded by the affiliative contexts (university clubs), involving an activity that occurs at a particular place and time, help better assimilate social features in a map-like format (Son et al., 2021). In contrast, Schwyck et al. (2023) found homophily based on trustworthiness interfered with memory for social network relationships, which parallels the effect traits had on relationships in the simple network. Taken together with the aforementioned findings, our results imply that multidimensional social features like contexts, or a combination of social attributes (Park et al., 2020, 2021; Tavares et al., 2015), can help bolster the underlying map-like structure through which social relationships are assimilated. In contrast, homophily can oftentimes be characterized based on one dimension (e.g., trustworthiness) and consequently wouldn’t require maplike learning mechanisms (Arzy & Kaplan, 2022). Furthermore, the multidimensional clubs aren’t valenced like traits, which could potentially explain why shared personality traits effects were more variable compared to contextual memory effects. Future work can distinguish if homophily based on multidimensional features (Block & Grund, 2014) exhibits more context-like effects on social network memory, and conversely whether valenced unidimensional contexts like a specific reward or punishment (Heald et al., 2023) exhibit more trait-like effects.

In context, our model was inspired by the distance-dependent Chinese Restaurant Process (dCRP; Blei & Frazier, 2011). The CRP itself has been used in cognitive science to describe how humans cluster items in an open-ended, nonparametric manner (Gershman & Blei, 2012; Gershman & Cikara, 2020), for example when studying social structure learning stereotype change (Gershman & Cikara, 2023), identifying and inferring latent causes (Gershman et al., 2015), as well as temporal community structures through random walks on graphs (Schapiro et al., 2013). In the classic CRP modeling approach, the CRP is used as a prior function that conditions an observation model, i.e., a likelihood function for making a-posteriori inferences. In our task, however, we developed a custom likelihood function that describes the distortion of memory for social structures towards certain semantic dimensions. However, we don’t propose a Bayesian approach to modeling, thus separating our approach to the CRP. Future studies may, however, find the Bayesian approach with an actual CRP to be fruitful when examining how the distortion of memory due to semantic context impacts the dynamics of choice behavior.

In sum, we find contexts support recall of friendship networks, while observing the valence of associated affective traits have a more variable effect on friendship recall. This phenomenon is observed in associative memory retrieval, as well as the implicit dimensions by which participants make similarity judgments. Taken together, our results highlight the nuances by which people structure social networks and give insights into how the same social network can be represented differently depending on the individual.

## ACKNOWLEDGMENTS

We thank the reviewers for providing constructive feedback on the paper.

## FUNDING INFORMATION

This research is supported by grants awarded to RK from the Spanish Science, Innovation, and University Ministry (PID2021-122338NA-I00), Valencian Community’s Program for the Support of Talented Researchers (CIDEGENT/2021/027), and Universitat Jaume I Research Advancement Plan (UJI-B2022-45). The funders had no role in study design, data collection and analysis, decision to publish, or preparation of the manuscript.

## AUTHOR CONTRIBUTIONS

R.K. and A.G. designed research; A.G. performed research; A.G. analyzed data; R.K. and A.G. wrote the paper.

## DATA AVAILABILITY STATEMENT

The data and code for the study can be found in https://github.com/Decision-and-Memory-Group-at-UJI/RelativeContributionsSocialCogMaps.

## Supplementary Material


