# Supplementary material for: The Relative Contributions of Traits and Contexts on Social Network Learning
Source: Open Mind (Camb). 2025 Sep 9;9:1506–27. doi: 10.1162/OPMI.a.31 (PMC12483571; doi:10.1162/OPMI.a.31)
Supplement: Supplementary file 1 [file opmi-09-1506-s001.pdf]

Supplementary Material

Semantic similarity Matrices of Trait and Context Labels

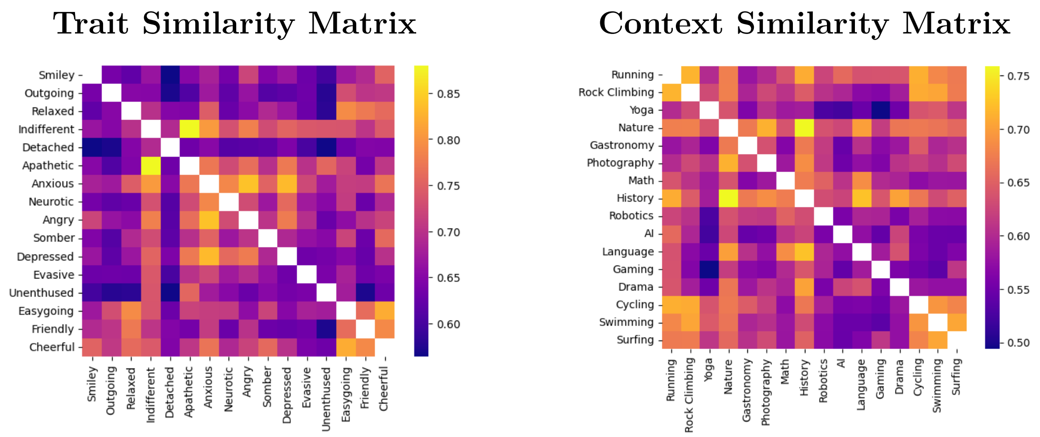

Figure S1: Heatmap of similarity matrices for either context labels or trait labels.

We computed pairwise semantic similarity matrices for both the personality trait labels and the context labels using GloVe word embeddings. These matrices are visualized as heatmaps (Fig. S1). The range of values in the similarity matrix for contexts was 0.265, while the range for personality traits was slightly higher at 0.316, indicating that, in terms of raw semantic distance, the trait labels were more separable than the context labels. Visually, both heatmaps show a comparable degree of clustering, with no clear indication that the trait labels formed more distinct or structured groupings than the context labels.

Representational Similarity Analysis of Behavioral Results

We performed a representational similarity analysis(RSA) to identify the weights of positive or negative valenced trait semantic similarity matrices, context semantic similarity matrices, and an indicator matrices for when student comparisons are made with central students in the network for predict-

ing the drag-and-drop results (i.e. the drag-and-drop distance matrix). When we related the regressor weights to the behavioral outcomes, i.e. friendship recall odds, we were not able to find any significant results. This indicates that representational similarity analysis did not have the same statistical power as our formal likelihood modeling approach we reported in the results. Speculatively, RSA may have failed due to linear and normality assumptions made when relating the theoretical RSA matrices to the behavioral drag-and-drop. The ability of our likelihood model to identify the nonlinear impact of cognitive biases, through the exponential gaussian kernel in the similarity matrix, may offer an explanation for its superior statistical power.

Qualitative Drag and Drop Figures

We qualitatively visualized the results of the drag-and-drop for each experiment to see if network structure and task conditions were reflected in the behavior. In Fig. S2A, we illustrate how on average, participants in Experiment 1 recover a 1 face planar graph, representative of the ground-truth graph, regardless of the condition. We visualize drag-and-drop behavior in the participant group that learned members in the correlated network condition in the top row of fig. S2B and C. The visualization shows that placements follow similar correlative behavior of the ground-truth GloVE semantic similarities. This figure shows how placements follow a specific axis of placement when using referential points (either using the word "smiley" or "running" as a baseline). This recapitulates the results seen statistically in fig 3B, where participants were able to learn multistep relationships significantly above chance. In the participant pool that learned members in an uncorrelated network, we show their drag-and-drop behavior does not have any correlated structure, as the task design imposes. Fig. S2D demonstrates how participants lose the clique structure of the network along the bridge node, but maintain the 2-face planar graph structure of the network. This reiterates the statistical result seen in fig. 4D.

Detail of the Leave-one-Out Cross Validation procedure

To compare the biased and unbiased models of participants' drag-and-drop data, we employed leave-one-out cross-validation (LOO-CV) at the level of dyads. For each participant, we iteratively held out a dyadic placement (i.e.,

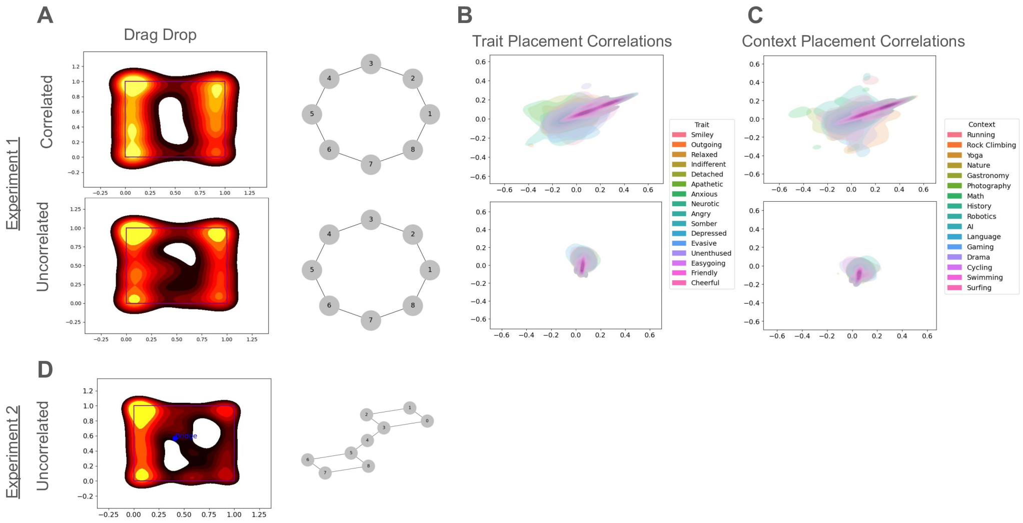

Figure S2: Illustrations of drag-and-drop behavior on average. (A-C) Experiment 1. (A) A heatmap of participants' drag-and-drop placement of network members shows the friendship network structure in both participant pools, where the color corresponds to placement count. (B) Participant placement clouds of network members with various personality traits, using the "Smiley" network member as a reference position, demonstrate consistency of placements with respect to semantic similarity when participants encoded correlated networks. (C) Participant placement clouds of network members with various contexts, using the "Running club" as a reference position, demonstrate similar placement consistency for participants encoding correlated networks. (D) Experiment 2: Heatmap of participants drag and drop results demonstrating reconstruction of the two faces of the group on average, with irresolution of the bridge connection between the two cliques, compressing the larger network structure into a single clique.

the relative distance between a pair of students), fit the model to the remaining placements, and evaluated the Kullback-Leibler divergence between the model's predicted friendship probabilities and the empirical drag-and-drop placement of the held-out dyad.

This procedure was repeated for both models, i.e. the Biased Model with separate β parameters for each attribute type (e.g., positive trait match, negative trait match, context, centrality), and the Unbiased Model: a shared β parameter across all attribute types.

The average held-out KL divergence across dyads was computed for each participant and model. We then conducted a paired-samples t-test across participants to assess whether the biased model better captured behavior (i.e., yielded lower held-out KL divergence) than the unbiased model.

Parameter Recovery

To assess the identifiability of our computational model of social memory biases, we conducted a parameter recovery analysis to ensure that the model fitting procedure can accurately distinguish between biased and unbiased generative processes. Specifically, we tested whether the model fitting and evaluation pipeline—based on minimizing the Kullback-Leibler (KL) divergence between predicted and observed drag-and-drop placements—correctly identifies the structure of the true underlying generative process. This was evaluated using the previously described Leave-one-out Cross Validation procedure.

We simulated synthetic participant data using the same likelihood model described in the main text. Two types of ground-truth agents were defined as either Biased agents, whose friendship placement decisions were guided by distinct biases across multiple feature dimensions (e.g., positively vs. negatively valenced traits, physical vs. nonphysical context of meeting, and centrality), and Unbiased agents, whose placements biases we're equivalent for all attributes.

For both agent types, ground-truth parameter sets (bias vectors β and dispersion parameter, α) were sampled from from log-normal distributions with reasonable ranges reflecting empirical data. The mean values are seen in Table S1. These parameters were then used to simulate drag-and-drop outputs. We subsequently attempted to recover the original model class (biased or unbiased) using the same model-fitting routine employed in the main analyses.

We compared the biased and unbiased models using leave-one-out (LOO) cross-validated KL divergence between the predicted and simulated drag-and-drop distributions. The key test statistic was the difference in LOO-KL divergence for each recovered model, with negative values indicating better fit by the biased model and positive values indicating better fit by the unbiased model.

For ground-truth biased agents, the model-fitting procedure successfully recovered the biased model as a significantly better fit (mean $T = -35.30$,

Parameter	μ	σ
Unbiased Agent		
α	0	1
β_{all}	-2	1
Biased Agent		
α	0	1
β_{positive}	-2	1
β_{negative}	1	1
β_{physical}	1	1
$\beta_{\text{non-physical}}$	1	1
$\beta_{\text{centrality}}$	0	1

Table S1: Table of parameters of generating distributions for parameters in the parameter recovery analysis.

$p < 0.001$). In contrast, for ground-truth unbiased agents, the unbiased model was preferred, with the biased model showing significantly worse fit (mean $T = 8.10$, $p < 0.001$). In Fig. S3, we show that for Biased agents, we could identify a uniqueness of positive biases. On the other hand, unbiased agents had no uniqueness in their biases.

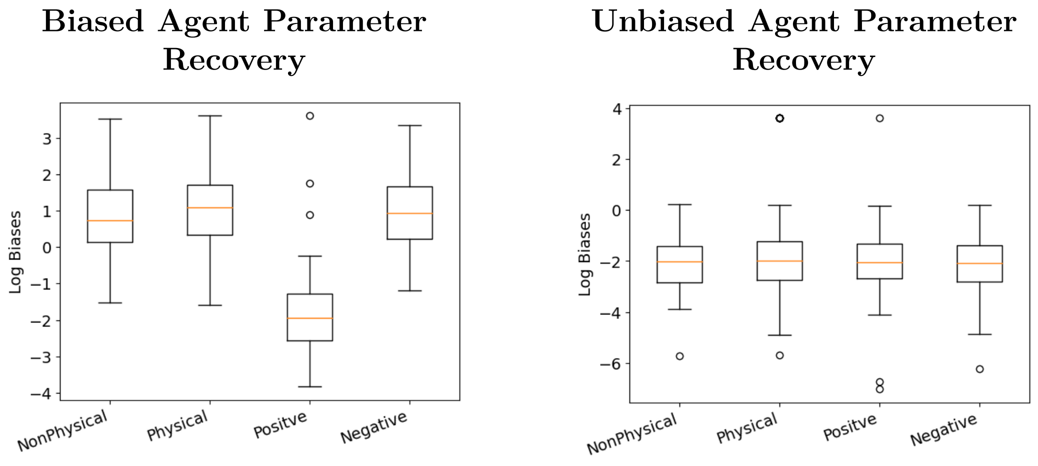

Figure S3: Boxplots of recovered parameters from the fitting procedure for each kind of agent.

These results demonstrate robust model identifiability: the fitting pro-

cedure is able to correctly infer whether behavior was generated by a process involving heterogeneous biases or a single undifferentiated factor. This suggests that the observed preference for the biased model in empirical data reflects meaningful structure in participants' cognitive representations rather than overfitting due to model complexity.
